# Intraoral Digital Impressions for Virtual Occlusal Records: Section Quantity and Dimensions

**DOI:** 10.1155/2016/7173824

**Published:** 2016-01-10

**Authors:** Eneko Solaberrieta, Asier Garmendia, Aritza Brizuela, Jose Ramon Otegi, Guillermo Pradies, Andras Szentpétery

**Affiliations:** ^1^Department of Graphic Design and Engineering Projects, University of the Basque Country (UPV/EHU), 48013 Bilbao, Spain; ^2^Department of Mechanical Engineering, University of the Basque Country (UPV/EHU), 20018 Donostia, Spain; ^3^Department of Prosthodontics and Occlusion, Faculty of Odontology, University of the Basque Country (UPV/EHU), 48940 Leioa, Spain; ^4^Department of Buccofacial Prostheses, Faculty of Odontology, Complutense University of Madrid, 28040 Madrid, Spain; ^5^Department of Prosthodontics, Faculty of Dentistry, University of Szeged, 6720 Szeged, Hungary

## Abstract

The purpose of this study was to locate the 3D spatial position mandibular cast and determine its occlusal contacts in a novel way by using an intraoral scanner as part of the virtual occlusal record procedure. This study also analyzes the requirements in quantity and dimensions of the intraoral virtual occlusal record. The results showed that the best section combination consists of 2 lateral and frontal sections, the width of this section being that of 2 teeth (24 mm × 15 mm). This study concluded that this procedure was accurate enough to locate the mandibular cast on a virtual articulator. However, at least 2 sections of the virtual occlusal records were necessary, and the best results were obtained when the distance between these sections was maximum.

## 1. Introduction

In recent years, the use of digital technology in dentistry has resulted in some important advances. The possibility of working in a virtual environment improves the diagnosis, planning, and treatment of any clinical case. Apart from this, working in a virtual environment shortens the time required for each procedure [1, 2]. Some recent developments have made the process and design of the final product more controllable and, therefore, more accurate. Besides this, the dental digital workflow can be closely monitored and tailored to the patient's requirements [3].

Since the use of intraoral scanning is becoming common practice in clinical work, several dental firms have centered their research efforts on intraoral scanners. Different studies have been carried out to assess the accuracy of the intraoral digital impression [4–7]. On the whole, the conclusions of these studies prove that, despite the excellent accuracy demonstrated by single-unit scans, the use of intraoral scanners in complete arch scans is not yet so accurate [8, 9]. Although most of the tested scanners showed similar values in single-unit scans, the results suggest that the inaccuracies obtained on complete arch scanning may contribute to inaccuracies in the final treatment [10, 11].

It is worth mentioning that, due to the high amount of best-fit alignment necessary to scan the complete arch, the main problem of scanning is the digitization of the complete arch. If an error is introduced in each best-fit alignment, the cumulative error is too high in the final part. This issue has been studied in depth over the last years by different authors [12–15]. For these studies reference models or stone casts (in vitro) were used, and, in order to obtain the reference, an industrial 3D scanner, a cone-beam computed tomography, or a coordinate measuring machine [16] was used. In one of these studies, significant differences were found between coating and noncoating scanners, and, for certain model materials, specific scanning errors for the system with parallel confocal microscopy were found [17, 18]. In summary, these studies concluded that, until the complete digital workflow is validated for extensive dentistry treatment, professionals should be very careful when using intraoral scanning and limit its use to shorter-spanned treatments.

After going through the most recent studies on intraoral scanners and, more specifically, on the virtual occlusal record with this type of scanners, it can be concluded that the conventional method of the interocclusal record for the positioning of the casts is now being replaced by intraoral digital impressions. Taking this fact into account, this study aimed to analyze the possibility of using the intraoral virtual occlusal record procedure as a novel way to locate the mandibular cast 3D spatial position in maximal intercuspidation and occlusal contacts, and in reference to its corresponding maxillary cast on a virtual articulator. Therefore, after studying the virtual occlusal record procedure using digitized plaster cast models [19, 20], the procedure was validated. To our knowledge, no study has assessed the validity of the virtual occlusal record procedure using an intraoral scanner directly on mouth (in vivo). Therefore, the aim of this study was to determine the validity and requirements of this procedure using an intraoral scanner. The requirements of this procedure when using an extraoral scanner were also determined in terms of best-fit alignments, sections, and dimension [21, 22]. This study tested the possibilities offered by 3 intraoral scanners in terms of accuracy and determined the requirements to carry out the virtual occlusal record procedure.

## 2. Material and Methods

To begin with, the IRB approval was obtained for a cross-sectional study with 4 participants scheduled for a diagnostic. In all cases, the study was carried out following two procedures: the conventional method and the virtual procedure. In both procedures, a Panadent articulator was used to locate the maxillary and mandibular casts.

**Figure 1:**
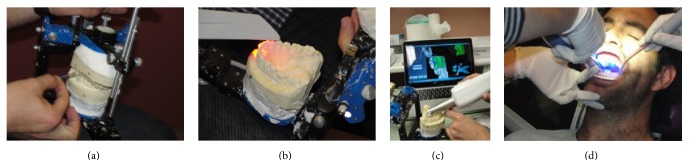
Conventional and virtual procedures. (a) Determination of occlusal contacts using Shimstock paper. (b) Scanning mandibular stone cast with Trios 3-Shape. (c) Scanning mandibular stone cast with Zfx Intrascan. (d) Scanning maxillary cast on participant with Lava Cos.

For the conventional procedure, the occlusal contacts were determined using a gold standard Shimstock paper (Arti-Fol metallic Shimstock-film 20 *μ*, Dr. Jean Bausch GmbH & Co.) and the articulating paper (8 *μ* Arti-Fol, Dr. Jean Bausch GmbH & Co.) was used to locate the occlusal contacts (Figure 1(a)).

Parallel to this conventional procedure, the digital workflow was performed. The maxillary and mandibular stone casts were scanned using an industrial 3-dimensional (3D) scanner (ATOS Compact Scan 5M, GOM GmbH). Then, the virtual occlusal records were taken using 3 different intraoral scanners: Lava Cos (3M Espe), Zfx Intrascan (MHT Technologies), and Trios 3-Shape (Phibo) (Figures 1(b)–1(d)). Besides this, using stone casts (in vitro) and directly on mouth (in vivo), the digitalization was finished using these intraoral scanners to scan the complete arches.

After the first phase, the accuracy of the input data on the virtual occlusal procedure was analyzed. There were 3 different input scan data items: maxillary cast, mandibular cast, and virtual occlusal record. Taking into account the characteristics of these inputs, the deviation of a single-unit scan and the deviation of a complete arch scan were determined using GOM Inspect (GOM Professional, v7.5, GOM mbH) reverse engineering software. The purpose of this calculation was to determine the accuracy of scanning systems in vitro and in vivo. The scanning obtained with the ATOS 3D scanner was taken as reference (accuracy of this system 0.03 mm).

After the second phase, the optimum combinations of the sections and dimensions were determined. The aim of this phase was to determine the sections of the occlusal record necessary to locate the mandibular cast on the correct position. As proposed by DeLong et al.'s study [19, 20], the correct position was determined by comparing the existing physical occlusal contacts and the determined virtual contacts. The reverse engineering software Geomagic (Geomagic Design X, 3D Systems) was then used to edit and process the virtual occlusal record. Four sets were mounted on a Panadent mechanical articulator (1801 AR Model PSH Articulator, Panadent Corp.) without any interocclusal record, and the physical occlusal contacts were checked virtually. Some references were taken with the complete virtual occlusal record and the virtual contacts were tested with the physical contacts. The physical occlusal contacts were located using 8 *μ*m articulating paper (8 *μ* Arti-Fol, Dr. Jean Bausch GmbH & Co.) and the contacts were verified with metallic gold standard polyester film (Arti-Fol metallic Shimstock-film 20 *μ*m, Dr. Jean Bausch GmbH & Co.).

Finally, once the required sections were determined, the size of the optimum virtual occlusal record for each section combination was established. In order to determine this size, a statistical analysis was carried out. The predictive values for 4 sets were calculated using the virtual occlusal records obtained with intraoral scanners. A diagnostic test was performed to measure the effectiveness of each procedure. The results of this diagnostic test provided valuable information regarding the probability of having or not having contacts. In this case, the virtual procedure was considered the diagnostic test (i.e., virtual occlusal recordings) and the reference diagnoses were the physical contacts obtained with the Shimstock paper [21]. Then, virtual contacts on the digital models were compared to the contacts obtained with the physical articulating paper. As described by DeLong et al. [19] the location of the contacts was based on anatomic regions and, as demonstrated by DeLong et al. [20], mean predictive values (mean PV) above 0.90 were considered accurate enough.

## 3. Results

To begin with, the resolution for the digitization systems used in this study was analyzed. This input variable was compared on the same molar using 3 different intraoral scanners as well as the extraoral scanner. In the case of the Lava Cos intraoral scanner, the high resolution option was applied. For the same digitized area, the quantity of triangles was calculated (Table 1).

**Figure 2:**
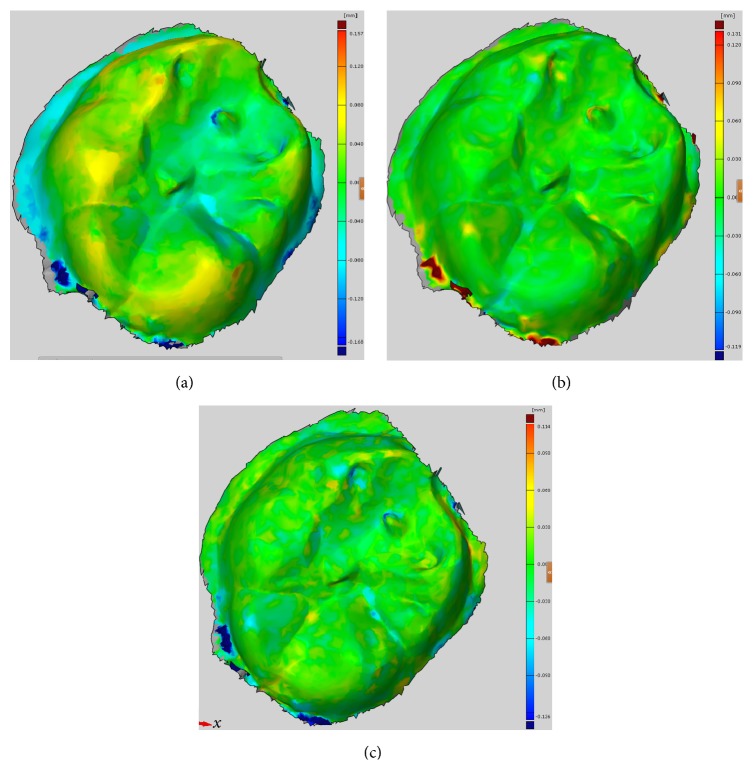
Deviation on a single-unit molar. (a) Zfx Intrascan. (b) Lava Cos. (c) Trios 3-Shape.

Afterwards, the deviations were calculated on the digitized stone casts (in vitro). The reference was the digital data obtained by ATOS industrial 3D extraoral scanner. The deviation in the single-unit scan was determined using GOM Inspect (GOM Professional, v7.5, GOM mbH) reverse engineering software. Zfx Intrascan, with 27.4 *μ*m, showed the highest mean deviation, and in terms of confidence intervals of the deviation the value of the 95% interval was *μ* (0.0181–0.0346). Lava Cos presented a mean deviation of 9.4 *μ*m and *μ* (0.0061–0.0146). Trios 3-Shape, with 8.8 *μ*m and *μ* (0.0051–0.0142), showed the least mean deviation (Figure 2).

**Figure 3:**
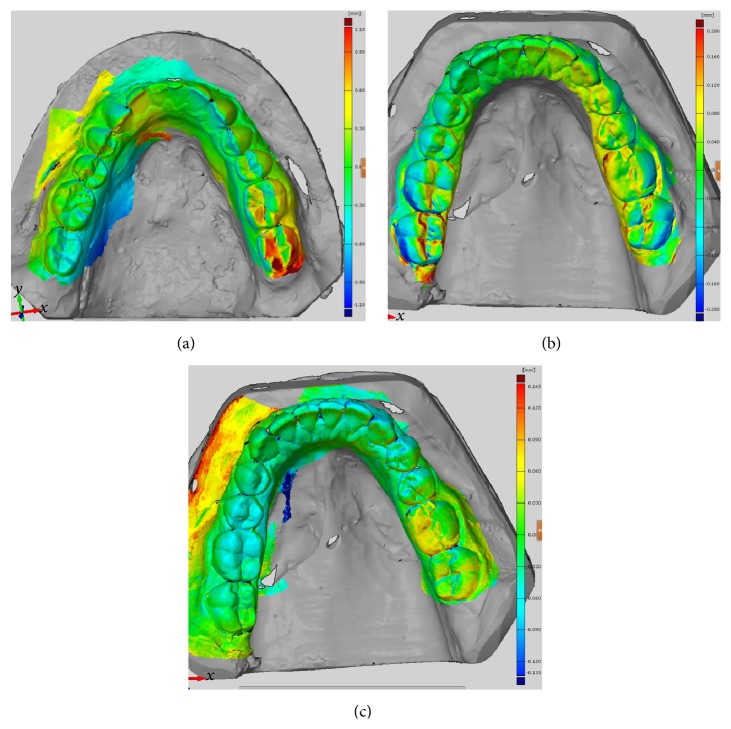
Deviation on complete arch. (a) Zfx Intrascan. (b) Lava Cos. (c) Trios 3-Shape.

The deviation for the complete arch was determined using the same software. The results were similar to the results obtained using a single-unit scan. The average deviation of a selection of 8 digitized stone casts (4 sets) was calculated. Zfx Intrascan, with 1.100 mm, showed the highest mean deviation. Lava Cos presented a mean deviation of 0.200 mm and *μ* (0.1032–0.3297). Trios 3-Shape, with 0.143 mm and *μ* (0.0551–0.2387), showed the least mean deviation (Figure 3).

Finally, the deviations on intraoral digitization (i.e., using an in vivo scan) were also calculated. The resolution was determined on a molar. Scanning both extra- and intraorally, ATOS 3D scanner obtained 15,740 triangles, Lava Cos obtained 20,592 triangles, and Trios 3-Shape obtained 13,896 triangles.

**Figure 4:**
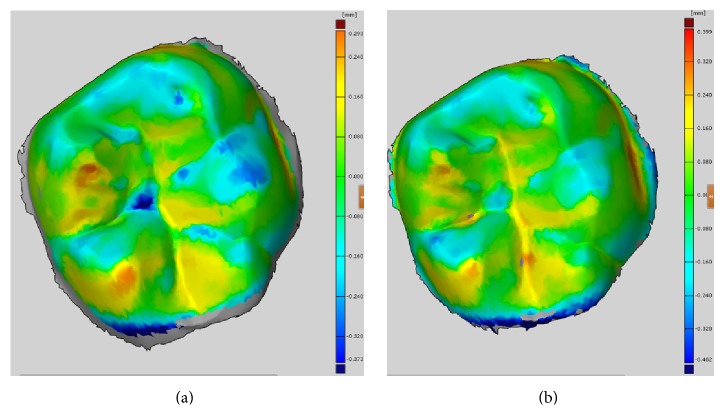
Deviation on a single-unit in vivo scan. (a) Lava Cos. (b) Trios 3-Shape.

The deviation in a single-unit in vivo scan was determined using Lava Cos and Trios 3-Shape intraoral scanners. Lava Cos presented a mean deviation of 78.0 *μ*m and *μ* (0.0282–0.0978). Trios 3-Shape, with 82.3 *μ*m and *μ* (0.0358–0.1183), presented a larger mean deviation (Figure 4).

**Figure 5:**
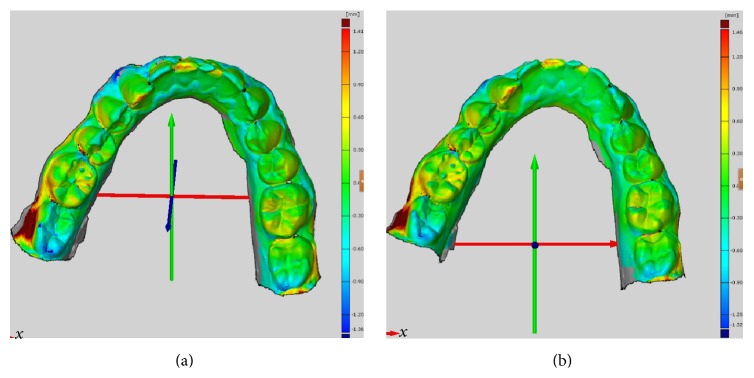
Deviation on complete arch. (a) Lava Cos. (b) Trios 3-Shape.

The deviation for the complete arch in vivo scan was then determined. Lava Cos showed a mean deviation of 0.2296 mm and *μ* (0.1152–0.3346). Trios 3-Shape presented a mean deviation of 0.2225 mm and *μ* (0.1284–0.2962) (Figure 5).

Having determined the accuracy of the input data (deviations and resolution), the requirements regarding quantity and dimensions of intraoral digital impressions for virtual occlusal records were determined. Since cumulative error of the Zfx Intrascan scanner was too high even for orthodontics treatment, this scanner was not used for this determination [21].

The predictive values with different sections were calculated for each set (TP: true positive, FP: true positive, TN: true negative, and FN: false negative). Positive predictive value (PV+) means probability of contact truly exists when diagnostic test is positive:

(1) $$\begin{array}{c} \left(\;PV+\;\right)=\frac{TP}{TP+FP}. \end{array}$$

Negative predictive value (PN−) means probability of contact is truly not present when diagnostic test is negative:

(2) $$\begin{array}{c} \left(\;PN-\;\right)=\frac{TN}{TN+FN}. \end{array}$$

Only the Lava Cos scanner offered the possibility to analyze 3 sections. The third set was analyzed using different section dimensions. The 3 contacts of this set were located using articulating paper, not virtually (FN = 3) (Table 2).

**Figure 6:**
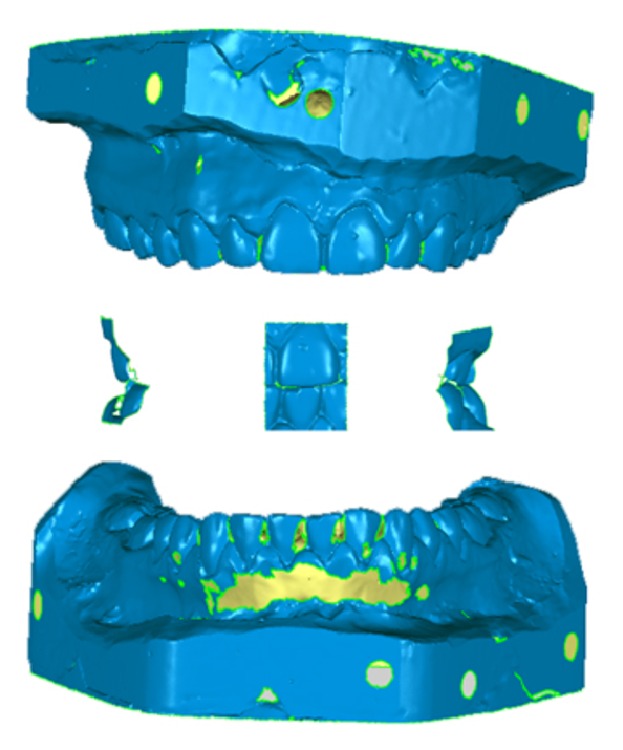
Combination of 2 lateral and frontal sections.

The resulting values showed that the best section combination was 2 lateral and frontal sections (Figure 6) with a 2-teeth-wide section (mean PV = 1.00). The other section combination that proved to be accurate enough was 2 lateral sections with a 3-teeth-wide section (mean PV = 0.91).

## 4. Discussion

Input data are of the utmost importance in the virtual occlusal record procedure. Straga [22] concluded that the less captures are used for the digitization, the more accurate is the determination of occlusal contacts. Therefore, when scanning the occlusal surface, in order to achieve a more accurate digitation, a minimum amount of captures must be made so as to have less best-fit alignments [8–10].

In terms of resolution there were no significant differences among different intraoral scanners compared with the ATOS scanner. However, due to the overlapped regions (best-fit alignment), there were some differences in terms of accuracy. The deviation was higher in the case of a complete arch than in the case of a single-unit scan. A comparison among the different intraoral scanners pointed out that the accuracy of the Trios 3-Shape was similar to that of the Lava Cos, while the least accuracy corresponded to the Zfx Intrascan system. Therefore, this scanner has not been used for the study of the virtual occlusal procedure. The results obtained by this study proved to be similar to those of other in vitro studies [8, 9, 17, 18]. Accuracy stayed between 8.8 and 9.4 *μ*m for a single-unit scan and between 0.143 and 0.200 mm for a complete arch scan. The accuracy values in vivo had not been studied in any previous research work. This study concluded that, in this case, the resulting values were higher. The single-unit scan showed some values between 78.0 *μ*m and 82.3 *μ*m. In the complete arch scan, these values stayed between 0.2225 mm and 0.2296 mm, showing a small increase in the complete arch deviation. The deviation magnitude difference between in vivo and in vitro scans or procedures is large, due to the movements, tongue, and spit. However, this deviation is not so large when the complete arch is scanned; this could be due to the compensation of best fit alignment.

This study has demonstrated that the resolution changed from stone models (in vitro) to mouth scanning (in vivo). The best-fit alignment issue is the same both intra- and extraorally. However, input data improve when working extraorally. That is, when the scanning is carried out directly on the patient's mouth the resolution and accuracy decrease [22]. Therefore, the accuracy of a single-unit and a complete arch scan is better when working in vitro. There are many reasons that account for this fact, among others, the brightness of the material, the patient's movement, and the presence or movement of the tongue. The learning curve of the intraoral scanner, especially in vivo, must be taken into consideration. In this study, an expert technician carried out in vivo scanning.

On the other hand, in vitro studies [1, 10, 17, 20] affirmed that there are more steps in the extraoral procedure where the deviation such as impression, production of the stone models, mounting on the mechanical articulator, and other steps could be introduced.

In terms of virtual occlusal record, the analysis of contacts was carried out as in previous studies [19–21]. To be able to locate the mandibular cast in relation to maxillary cast, the software of these intraoral scanners requires 2 or 3 sections. This study concluded that 3 sections of 24 mm width and 15 mm height were the best combination, with the distance among the sections being as large as possible. This section could be obtained with 2-3 successive image captures with any of these intraoral scanners and larger sections introduce more deviation due to the fact that more “best-fit alignments” are used on the scanning phase. The second best combination consisted of 2 lateral sections (36 mm wide and 15 mm high). This is the combination recommended by Trios 3-Shape.

In summary, the literature review confirmed the influence of different variables on the virtual occlusal record procedure [21, 22]. The deviation of the virtual occlusal record procedure depends on a variety of parameters such as scan quality (accuracy), best-fit alignment, and the amount of sections and dimensions of the virtual occlusal records.

## 5. Conclusions

Intraoral virtual occlusal recording is a valid procedure to locate a mandibular cast on a virtual articulator. The contacts observed with this procedure were accurate enough. Moreover, virtual contacts provided more objective and meaningful information. However, due to the cumulative error, knowing the deviation of each alignment (best-fit operation or algorithm) is certainly useful.

Lava Cos and Trios 3-Shape intraoral scanners showed similar characteristics, both of them being good enough to carry out this procedure. There were no significant differences between them in terms of accuracy. The main difference between these intraoral scanners was in terms of patient comfort. The Lava Cos requires coating and has a comfortable small tip, whereas the Trios 3-Shape scanner has a larger tip and is much faster.

Another main conclusion was that the best results regarding the virtual occlusal record sections were obtained when the distance between the sections was maximum. Depending on the system, and although each section does not have to be wide (to avoid best-fit alignments), 2 or 3 sections can be required. The resolution and accuracy were similar in Lava Cos and Trios 3-Shape scanner. However, this second intraoral scanner only permits 2 sections as occlusal record. In this case, the most important point is that the distances between these sections have to be as large as possible. This explains why the molar part is usually scanned.

Since the real advantages of this procedure can only be proven in vivo, more studies must be carried out in the future in in vivo conditions.

## Figures and Tables

**Table 1:** Quantity of triangles for the same molar for different scanning systems.

Scanner	ATOS	3-Shape	Zfx	Lava Cos	Lava Cos (high)

Triangle quantity	19.771	16.668	18.518	21.860	62.228

**Table 2:** Statistical analysis for intraoral virtual occlusal record.

3rd set-virtual occlusal record	FP	TP	FN	TN	PV+	PV−	Mean PV
Lava Cos (section, width)							
2 lateral sections, 1 tooth	2	4	1	3	0.67	0.75	0.71
2 lateral sections, 2 teeth	0	6	1	3	1.00	0.75	0.87
2 lateral sections, 3 teeth	1	5	0	3	0.83	1.00	**0.91**
2 lateral and frontal sections, 1 tooth	0	5	1	3	1.00	0.75	0.87
2 lateral and frontal sections, 2 teeth	0	6	0	3	1.00	1.00	**1.00**
2 lateral and frontal sections, 3 teeth	1	5	1	3	0.83	0.75	0.79
2 lateral sections, 5 teeth	1	4	2	3	0.80	0.60	0.70
Complete occlusal record	1	3	2	3	0.75	0.60	0.67
